# Comparison of onset time, duration of action, and intubating conditions after cisatracurium 0.15 mg/kg in young and elderly patients

**DOI:** 10.1186/s12871-022-01881-5

**Published:** 2022-11-07

**Authors:** Matias Vested, Camilla Meno Kristensen, Pernille Pape, Malene Vang, Mian Hartoft, Caroline Hjelmdal, Lars Simon Rasmussen

**Affiliations:** 1 Department of Anesthesia, Centre of Head and Orthopedics, Rigshospitalet, University of Copenhagen, Inge Lehmanns Vej 6, Section 6011, DK-2100 Copenhagen, Denmark; 2 Department of Clinical Medicine, University of Copenhagen, Copenhagen, Denmark

**Keywords:** Neuromuscular blocking agents, Onset time, Duration of action, Intubating conditions, Elderly patients

## Abstract

**Introduction:**

Tracheal intubation during anesthesia can be facilitated by the neuromuscular blocking agent cisatracurium. However, limited data exists about onset time, duration of action and effect on intubating conditions in elderly patients above 80 years of age. We hypothesized that elderly patients would present a longer onset time and duration of action compared to younger adults.

**Methods:**

This prospective observational study included 31 young (18–40 years) and 29 elderly (≥ 80 years) patients. Patients were given fentanyl 2 μg/kg and propofol 1.5–2.5 mg/kg for induction of anesthesia and maintained with remifentanil and propofol. Monitoring of neuromuscular function was performed with acceleromyography. Primary outcome was onset time defined as time from injection of cisatracurium 0.15 mg/kg (based on ideal body weight) to a train-of-four (TOF) count of 0. Other outcomes included duration of action (time to TOF ratio ≥ 0.9), intubation conditions using the Fuchs-Buder scale and the Intubating Difficulty Scale (IDS), and occurrence of hoarseness and sore throat postoperatively.

**Results:**

Elderly patients had significantly longer onset time compared with younger patients; 297 seconds (SD 120) vs. 199 seconds (SD 59) (difference: 98 seconds (95% CI: 49–147), *P* < 0.001)). Duration of action was also significantly longer in elderly patients compared with younger patients; 89 minutes (SD 17) vs. 77 minutes (SD 14) (difference: 12 minutes (95% CI: 2.5–20.5) *P* = 0.01)). No difference was found in the proportion of excellent intubating conditions (Fuchs-Buder); 19/29 (66%) vs 21/31 (68%) (*P* = 0.86) or IDS score (*P* = 0.74). A larger proportion of elderly patients reported hoarseness 24 hours postoperatively; 62% vs 34% *P* = 0.04.

**Conclusion:**

In elderly patients cisatracurium 0.15 mg/kg had significantly longer onset time and duration of action compared with younger patients. No difference was found in intubating conditions at a TOF count of 0.

**Trial registration:**

Clinicaltrials.gov (NCT04921735, date of registration 10 June 2021).

## Background

The neuromuscular blocking agent cisatracurium can be administered to facilitate tracheal intubation, however little is known about its onset time, duration of action and influence on intubating conditions in elderly patients above 80 years, a rapidly growing surgical population [1].

Cisatracurium is a non-depolarizing neuromuscular blocking agent (NMBA) which is primarily metabolized by Hofmann’s elimination, and therefore regarded as almost non-dependent on renal and liver function [2, 3]. Thus, in elderly patients, cisatracurium may be a rational choice for neuromuscular blockade, since many elderly are characterized by a decrease in liver and renal function, as well as a reduction in cardiac output [4]. Furthermore, it has been suggested that cisatracurium has less variability in duration of action compared with other NMBAs such as rocuronium [5]. In young adults cisatracurium 0.1 mg/kg has an onset time of approximately 180 seconds and a duration of action of 40 minutes [6]. However, as elderly patients are characterized by increased proportions of fatty tissues and reduced total body water [7] these changes may influence onset time and duration of action of NMBAs such as cisatracurium [8].

It is possible to assess onset time and duration of action of cisatracurium by monitoring the neuromuscular response from the adductor pollicis muscle by train-of-four (TOF) stimulation at the ulnar nerve. In elderly patients especially, a prolonged duration of action of NMBAs is associated with an increased risk of muscle weakness, double or blurred vision, impaired pulmonary function, and a higher risk of postoperative respiratory complications [9–12]. It is hence important to know both the duration of action and onset time of cisatracurium in the elderly patients above 80 years of age.

The aim of this study was to determine the onset time, duration of action and intubation conditions after administration of cisatracurium 0.15 mg/kg in elderly patients with an age of 80 years and older, and in younger patients aged 18–40 years. The hypothesis was that cisatracurium had a longer onset time and duration of action in elderly patients compared with younger patients.

## Methods

The data management in this prospective observational study was approved by the Danish Data Protection Agency (25 March 2021 - P-2021-251). The Danish Medicines Agency (5 November 2020, journal number 2020103276) and The Regional Committee on Health Research Ethics, The Capital Region, Regionsgården Kongens Vænge 2, DK- 3400 Hillerød, Denmark (30 October 2020, journal number 20070639) deemed this study to be exempted from ethical approval according to Danish legislation because of the observational study design with no intervention. This decision was made with reference to Danish law (komitéloven § 1, stk. 4)“ and “The Scientific Ethics Committee (30 October 2020, journal number 20070639) deemed this study to be exempted from ethical approval. The study was conducted in agreement with the Declaration of Helsinki and written informed consent was obtained from all patients. The study was registered at Clinicaltrials.gov (NCT04921735, date of registration 10 June 2021) prior to enrolment of patients. Data was stored in a REDCap database and the manuscript adheres to CONSORT guidelines. Patients were included from the Department of Anesthesia, Centre of Head and Orthopedics, Rigshospitalet, University of Copenhagen, Denmark.

Patients scheduled for elective surgery under general anesthesia, with an expected duration of anesthesia > 1 hour, with planned intubation and use of cisatracurium were included if they were between 18 and 40 years of age or above 80 years of age, and had an American Society of Anesthesiologists (ASA) physical status classification I to III. Patients were excluded if they had known allergy to cisatracurium, neuromuscular disease interfering with monitoring of neuromuscular function, surgery in the prone position or indication for rapid sequence induction.

### Anesthesia

The patients had an intravenous catheter inserted in a vein of the forearm opposite to the neuromuscular monitor and were monitored with electrocardiogram, non-invasive blood pressure, pulse oximetry and core temperature. Fentanyl 2 μg/kg and propofol 1.5–2.5 mg/kg were administered for induction of anesthesia after preoxygenation. After obtainment of a stable neuromuscular signal 2 min after calibration, cisatracurium 0.15 mg/kg was injected over 5 seconds. The dose of cisatracurium was based on ideal body weight, calculated in kg as height (cm) minus 105 for women and height (cm) minus 100 for men, or actual body weight, whichever the lowest. In the medicine room the study medicine was prepared under double control by two investigators.

When the train-of-four (TOF) count was 0 tracheal intubation was done and intubating conditions were assessed by the Fuchs-Buder scale [13] and the Intubating Difficulty Score (IDS) [14]. Also use of a videolaryngoscope or a stylet was recorded. Patients were mechanically ventilated targeting normocapnia after tracheal intubation. Infusions of propofol of approximately 5 mg/kg/hour and remifentanil 0.25–0.5 μg/kg/min maintained anesthesia. Administration of ephedrine or phenylephrine was recorded from induction of anesthesia until tracheal intubation. Core temperature above 35 °C and a peripheral skin temperature above 32 °C was secured by an upper body forced air warming system. Extubation was done upon full recovery from neuromuscular block (TOF > 0.9, not normalized data). Neostigmine (30–50 μg/kg) was administered if spontaneous recovery of the neuromuscular blockade did not occur. Patients were given standard postoperative pain treatment, comprising opioids, NSAIDs and paracetamol.

Patients rated occurrence of hoarseness or sore throat on a numeric rating scale (0–10) 24 h postoperatively. Patients who experienced either or both repeated the ratings on the third day postoperatively.

### Neuromuscular monitoring

Neuromuscular function was monitored according to the established guidelines from Good Clinical Research Practice (GCPR) for pharmacodynamic neuromuscular studies [15]. The acceleromyograph TOF-Watch SX was connected to a computer for collection of neuromuscular data (Version 2.5 INT 2007; Organon, The Netherlands). Two ECG electrodes (Ambu® BlueSensor N; Copenhagen Denmark) were placed over the ulnar nerve on the wrist after cleaning the skin with a disinfectant wipe. The acceleration transducer was placed on the thumb with a hand adaptor and upon loss of eyelash reflexes the TOF Watch SX was started. Two TOF nerve stimulations were given, followed by tetanic stimulation with 50 Hz for 5 seconds. Calibration was performed with the CAL button and neuromuscular function was monitored by TOF stimulation (2 Hz for 1.5 s) every 15 s. Neuromuscular data were pseudo-anonymized and stored on a drive.

### Endpoints

The primary outcome was onset time, defined as time from the start of injection of cisatracurium to a train-of-four (TOF) count of 0. Secondary outcomes included duration of action defined as time from start of the injection of cisatracurium to a TOF ratio ≥ 0,9, intubation conditions evaluated by the IDS [14] and Fuchs-Buder scale [15], occurrence of sore throat and postoperative hoarseness, and the administration of ephedrine or phenylephrine from induction of anesthesia until tracheal intubation.

### Statistical analysis

We used SAS Studio 3.8 software. Patient characteristics and outcomes were reported as means with standard deviations (SD), medians with interquartile ranges (IQR) or counts with frequencies. A T-test was used for comparison of parametric data, while nonparametric data were compared with a Mann-Whitney U test, and proportions were compared with Chi Square or Fisher’s exact test. Mean differences were calculated and reported with 95% confidence intervals. A *P* value < 0.05 was considered statistically significant.

The sample size calculation was based on our primary hypothesis. From previously conducted studies [16, 17], we estimated a 45 seconds standard deviation for the onset time. We estimated that a 30 seconds difference in onset time was the least clinically relevant difference between the young patients (18–40) and the elderly (≥ 80). Based on a sample size analysis we calculated that 30 patients in each group would allow us to detect this difference with a power of 80 and 5% risk of type 1 error.

## Results

**Fig. 1:**
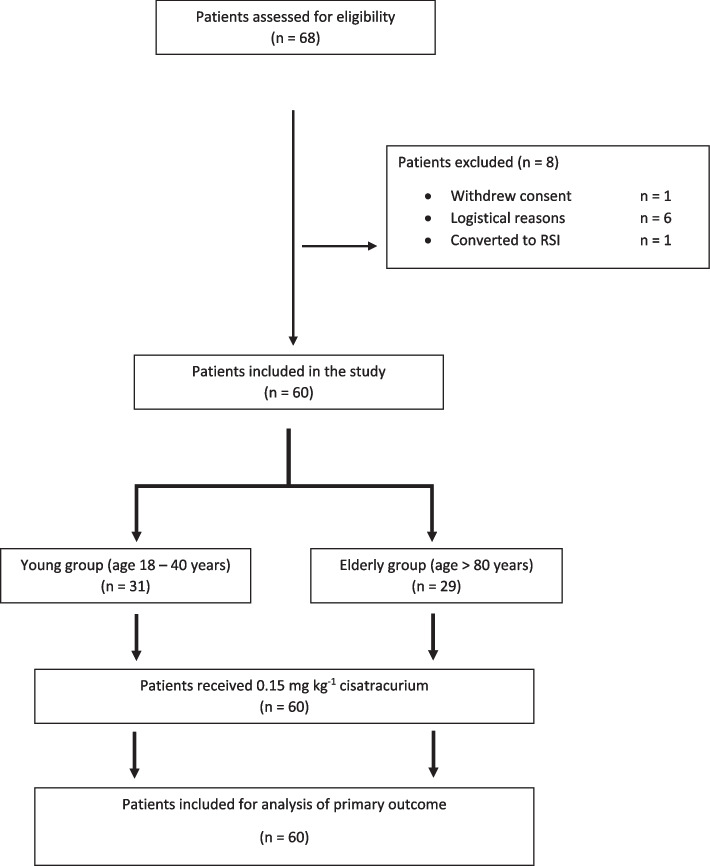
Flowchart

A total of 68 patients were eligible, of which one patient withdrew consent, six patients were excluded due to logistical reasons and one patient had an indication for a rapid sequence induction (Fig. 1). A total of 60 patients were included from July 5 2021 to December 19 2021 (Table 1).

**Table 1:** Baseline characteristics of patients (young and elderly).administered cisatracurium 0.15 mg/kg. *n* = 60

	Young	Elderly
n	31	29
Age years	30 (6.9)	84 (4)
Sex M/F	9/22	7/22
BMI kg/m^2^	24.8 (4)	24.9 (4)
ASA I/II/III	15/15/0	0/16/13
Daily medicine		
Diuretics	0	13 (45%)
Magnesium	0	1 (3%)
Comorbidity		
Diabetes	0	2 (7%)
Hypertension	0	20 (69%)
Heart disease	1 (3%)	5 (17%)

Data presented as count and frequencies (%) or mean and standard deviation (SD)

Elderly patients had a significantly longer onset time compared with younger patients; 297 seconds (SD 120) vs. 199 seconds (SD 59) (difference: 98 seconds (95% CI: 49–147), *P* < 0.001)). Duration of action was also significantly longer in elderly patients compared with younger patients; 89 minutes (SD 17) vs. 77 minutes (SD 14) (difference: 12 minutes (95% CI: 2.5–20.5) *P* = 0.01)) (Table 2).

**Table 2:** Intraoperative data including onset time and duration of action for cisatracurium 0.15 mg/kg in young and elderly patients

	Young	Elderly	Difference with 95%CI	*P*-value
n	31	29		
Onset time, seconds Time to TOF 0	199 (59)	297 (120)	98 (49 to 147)	< 0.001
Duration of action, minutes Time to TOF 0.9	77 (14) *n* = 24	89 (17) *n* = 26	12 (2.5 to 20.5)	0.01
Time used for intubation, seconds^a^	92 (74)	115 (157)	23 (−86 to 40)	0.46
Duration of anesthesia, minutes	188 (137)	189 (67)	–	–
Duration of surgery, minutes	118 (123)	108 (57)	–	–
Administration of supplemental cisatracurium after intubation	2 (6%)	1 (3%)		
Administration of neostigmine	5 (17%)	1 (3%)		
Administration of ephedrine or phenylephrine before TOF 0	0	12 (43%)		
Type of surgery			–	–
Plastic/breast	24	19		
Orthopedic	7	10		
Use of inhalational anesthesia during surgery	1 (3%)	0	–	–

Data presented as count and frequencies (%) or mean and standard deviation (SD)

In seven patients the duration of action was not determined due to administration of neostigmine, administration of supplemental doses of cistracurium or monitoring difficulties

^a^From taking the laryngoscope until tracheal intubation verified by capnography

No difference in IDS score was found comparing elderly patients with younger patients; median 0 (IQR: 0–2) vs. median 0 (IQR: 0–1) (*P* = 0.74). IDS > 0 was seen in 16 (55%) elderly and 18 (58%) younger patients (*P* = 0.82) (Table 3). No difference was found in the proportion of excellent intubating conditions (Fuchs-Buder); 19/29 (66%) vs 21/31 (68%) (*P* = 0.86) (Table 4).

**Table 3:** Tracheal intubating conditions assessed after administration of cisatracurium 0.15 mg/kg in young and elderly patients

	Young	Elderly	Difference (%) with 95% CI	*P*-value*
n	31	29		
Excellent intubating conditions (Fuchs-Buder)	21 (68%)	19 (66%)	2 (−22 to 26)	0.86
Use of video laryngoscope	5 (16%)	13 (45%)	29 (6 to 51)	0.02
Use of stylet	6 (19%)	15 (52%)	32 (9 to 55)	0.01
Intubating Difficulty Score (IDS)	0 (0–1)	0 (0–2)		0.74**
IDS > 0	18 (58%)	16 (55%)	3 (−28 to 22)	0.82
Sore throat 24 hours postoperatively	7 (24%)	5 (19%)	5 (−17 to 27)	0.66
Hoarseness 24 hours postoperatively	10 (34%)	16 (62%)	27 (2 to 53)	0.04
Sore throat 72 hours postoperatively	4 (14%)	2 (8%)	5 (−11 to 22)	0.53
Hoarseness 72 hours postoperatively	4 (17%)	5 (17%)	1 (−20 to 20)	0.96

Data presented as count and frequencies (%) or mean and standard deviation (SD)

IDS presented as median and interquartile range (IQR)

*CI* Confidence Interval

*Chi Square test

**Mann Whitney U test

**Table 4:** Tracheal intubating conditions (Fuchs-Buder) assessed after administration of cisatracuronium 0.15 mg/kg in young and elderly patients

	Young	Elderly	*P*-value*
n	31	29	
**Vocal cords position**			0.11
1 Abducted	28 (90%)	22 (76%)	
2 Intermediate	2 (7%)	7 (24%)	
3 Closed	1 (3%)	0	
**Vocal cords movement**			0.37
1 None	30 (97%)	28 (97%)	
2 Moving	0	1 (3%)	
3 Closing	1 (3%)	0	
**Reaction to intubation: Movement**			0.71
1 None	28 (90%)	26 (90%)	
2 Slight	1 (3%)	2 (7%)	
3 Vigorous	2 (7%)	1 (3%)	
**Reaction to intubation: Coughing**			0.7
1 None	28 (90%)	27 (93%)	
2 Slight	3 (10%)	2 (7%)	
3 Sustained	0		
**Laryngoscopy: Jaw relaxation**			0.32
1 Relaxed	25 (81%)	27 (93%)	
2 Not fully	5 (16%)	2 (7%)	
3 Poor	1 (3%)	0	
**Laryngoscopy: Resistance to laryngoscope**			0.32
1 None	28 (90%)	27 (93%)	
2 Slight	1 (3%)	2 (7%)	
3 Reactive	2 (7%)	0	

Data presented as count and frequencies (%),

*Chi-Square

Twenty-four hours postoperatively no difference was found in the occurrence of sore throat (*P* = 0.66), but hoarseness which was reported among a significantly larger proportion of the elderly; 16/29 (62%) vs 10/31 (34%) (*P* = 0.04). No differences were found in postoperative hoarseness or sore throat 72 hours postoperatively (Table 3).

No younger patients required administration of ephedrine or phenylephrine, as opposed to 12 (43%) elderly patients (Table 2).

## Discussion

We found that elderly patients had a significantly longer onset time (mean 297 vs. 199 seconds) and duration of action (mean 89 vs. 77 minutes) of cisatracurium 0.15 mg/kg compared with younger adults. However, no difference was found in intubating conditions using the IDS score or the Fuchs-Buder scale when the TOF count was 0.

Excellent intubating conditions were reported in 66% of the elderly patients after administration of cisatracurium 0.15 mg/kg, and no difference was seen between young and elderly. However, a videolaryngoscope was used in 45% of the elderly compared with 16% of the younger patients (*P* = 0.02) which may have influenced the assessment of tracheal intubating conditions, and this is a limitation in our study. Thus, videolaryngoscopy was used as first choice more often in the elderly but no difference was found in IDS scores including the number of attempts. Other limitations included the assessment of tracheal intubating conditions by multiple clinicians, possibly introducing interrater variability, as well as assessment of intubating conditions using the IDS score which is based on the employment of direct laryngoscopy. However, previous studies of intubating conditions have successfully used the IDS comparing direct laryngoscopy with the use of a videolaryngoscope [18]. The strength of our study lies in the neuromuscular monitoring, carried out according to research guidelines and managed by few, specifically trained, investigators providing exact data on onset time and duration of action [15].

Other studies have compared onset time and duration of action of cisatracurium in younger and elderly patients. Cisatracurium 0.15 mg/kg administered during total intravenous anesthesia resulted in a similar onset time; 250 seconds and 260 seconds in young and elderly patients, respectively [5]. Administration of cisatracurium 0.1 mg/kg during inhalational anesthesia resulted in a prolonged onset time in the elderly patients (240 vs 180 seconds (*P* < 0.01)) in one study [19] whereas a similar study reported no difference in onset time approximately 310 seconds vs 250 seconds in elderly and younger patients, respectively [3]. None of the three studies reported differences in duration of action comparing the young with the elderly patients [3, 5, 19]. When interpreting these previous data in relation to our results, it is important to underline that the two studies administered smaller doses of cisatracurium (0.1 mg/kg) [3, 19] and their designs compared age groups with a wider range i.e. the elderly patients had a mean age of approximately 70 years [3, 5, 19].

The observed difference in onset time may be related to an increased circulation time and reduction in cardiac output as suggested in previous studies on elderly patients [3, 20]. The pharmacokinetic profile of cisatracurium however, has been reported to be only minimally affected in elderly patients [3] and some studies report wide standard deviations of the duration of action of cisatracurium [3, 19]. It is possible that we were able to detect a difference in duration of action because of our study design, involving two age groups with a relatively large difference. The mean difference in duration of action of 12 minutes however, had a 95% confidence interval ranging from 2.5 to 20.5 minutes, meaning that the difference may be only 2.5 minutes, which is of minor clinical relevance.

The occurrence of excellent intubating conditions in two thirds of the elderly in our study is in contrast to a recent study on elderly patients administered rocuronium 0.6 mg/kg where excellent intubating conditions occurred in less than 30% [21]. In both studies intubating conditions were assessed upon a TOF count of 0 monitored at the ulnar nerve. Also, a recent study reports that increasing the dose of rocuronium from 0.6 to 1.0 mg/kg was associated with a larger proportion of excellent tracheal intubating conditions when TOF count was 0 [22]. The difference in intubating conditions may reflect the different muscle sensitivity towards NMBAs [23] and the difference in potency between cisatracurium and rocuronium. It is possible that a TOF count of 0 detected at the ulnar nerve after cisatracurium 0.15 mg/kg provides better intubating conditions than rocuronium 0.6 mg/kg.

Cisatracurium is a potent non-depolarizing NMBA with a longer onset time than less potent NMBAs such as rocuronium, which in the elderly has an onset time of 135 seconds [8]. In elderly patients who have an increased risk of prolonged duration of action of NMBAs [1] however, cisatracurium could be a rational choice due to its almost organ independent elimination [3]. In relation to this, one important finding from this study was that both onset time and duration of action were prolonged in the elderly and offered no advantage compared to rocuronium 0.6 mg/kg where duration of action is around 80 minutes [8]. In addition, it is possible to reverse a rocuronium-induced NMB with sugammadex within few minutes, which is not possible for cisatracurium [24]. Finally, the dose of cisatracurium was based on ideal body weight and it is important to emphasize that cisatracurium 0.15 mg/kg based on actual body weight would cause an even longer duration of action.

Regardless of the type of NMBA administered, this study illustrates the importance of objectively monitoring the neuromuscular blockade especially in the elderly, both for guidance of optimal timing for intubation, but also to reduce the risk of postoperative residual blockade [1, 25].

As an alternative to cisatracurium, a bolus of remifentanil can be administered for facilitating tracheal intubation. However, in a study on elderly patients excellent intubating conditions were found in less than 40% of the patients after administration of remifentanil 2 μg/kg despite the use of a videolaryngoscope in 50% of cases [21]. Also, it is relevant to emphasize that tracheal intubation was commenced already two minutes after administration of the study drug.

In conclusion, this study found that cisatracurium 0.15 mg/kg had a significantly longer onset time and duration of action in elderly compared with younger patients. No difference was found in intubating conditions at a TOF count of 0.

## Data Availability

The datasets used and analysed during the current study are available from the corresponding author on reasonable request.
